# Clients’ Experience and Satisfaction of Utilizing Healthcare Services in a Community Based Health Insurance Program in Bangladesh

**DOI:** 10.3390/ijerph15081637

**Published:** 2018-08-02

**Authors:** Abdur Razzaque Sarker, Marufa Sultana, Sayem Ahmed, Rashidul Alam Mahumud, Alec Morton, Jahangir A.M. Khan

**Affiliations:** 1 Health Economics and Financing Research, icddr,b, Dhaka 1212, Bangladesh; sayemahmed@icddrb.org; 2 Department of Management Science, University of Strathclyde, Glasgow G1 1XQ, UK; alec.morton@strath.ac.uk; 3 Nutrition and Clinical Services Division, icddr,b, Dhaka 1212, Bangladesh; marufa@icddrb.org; 4 School of Health and Social Development, Deakin University, Burwood, Melbourne, VIC 3125, Australia; 5 Department of Learning, Informatics, Management and Ethics (LIME), Karolinska Institutet, SE-171 77 Stockholm, Sweden; Jahangir.Khan@lstmed.ac.uk; 6 Health Economics and Policy Research, University of Southern Queensland, Toowoomba, QLD 4350, Australia; rashed.mahumud@usq.edu.au; 7 Department of Clinical Sciences, Liverpool School of Tropical Medicine, Liverpool L3 5QA, UK

**Keywords:** health insurance, healthcare services, satisfaction, informal worker, Bangladesh

## Abstract

*Background*: Community-based health insurance is recognized as a promising tool for health system improvement for low-income people that improves the health status of enrolees and enhances productivity and labor supply. The experience and opinion of the clients who utilized health services through the insurance scheme are important for improving healthcare services, shaping health policies and providing feedback on the quality, availability, and responsiveness of healthcare services. However, studies focusing on clients’ satisfaction provided by the health insurance scheme are still limited globally. *Objective*: To address this knowledge gap, this current study attempted to measure the degree of clients’ satisfaction towards healthcare services and insurance scheme, based on their experience of health care which will serve the future reference point to implement potential quality improvement initiatives of community-based health insurance program. *Methods*: A cross-sectional household survey was conducted within the catchment area of a community-based health insurance pilot program named Labor Association for Social Protection (LASP) during April–June 2014 to compare the evaluation of healthcare services provided by LASP scheme. In the descriptive analyses, the characteristics of the study participants were presented regarding frequency and the percentages with 95% confidence interval. Spearman correlation analysis was conducted between the satisfaction score of each indicator and overall satisfaction score; multivariate linear regression analysis was used to identify the factors associated with overall health scheme satisfaction. *Results*: The overall satisfaction mean score was 4.17 ± 0.04 (95% CI: 4.08–4.26) out of 5.00. The most satisfied domains were related to the diagnostic services (4.46 ± 0.98), explanation about the prescribed medicine (4.23 ± 0.81), the surrounding environment of healthcare facility (4.21 ± 0.70) and the behavior of health personnel toward clients (4.18 ± 0.73). *Conclusions*: Our study observed that the overall satisfaction level towards health services is quite favorable, but satisfaction scores can still be improved. These findings could contribute towards developing and designing the healthcare services packages of community-based health scheme which is in line with the health care financing strategy of Bangladesh as well as the recommendation of the World Health Organization for developing social health insurance as part of path to Universal Health Coverage.

## 1. Introduction

The World Health Organization has recommended that all United Nation member achieve universal health coverage (UHC) status by 2030 as a part of the recent Sustainable Development Goals, as half of the world’s population still unable to obtain essential health services [1]. According to the UHC theme, all individuals and communities who need health services should receive them without suffering financial hardship. Removing financial hardship is particularly crucial for developing countries like Bangladesh, where out-of-pocket (OOP) is the main payment strategy for healthcare, and the OOP share has been increased alarmingly from 55.9% in 1997 to 67% in 2015 [2]. Moreover, this scenario becomes more vulnerable considering the low-income people and as a consequence, many households experience catastrophic economic burden and fall into poverty in Bangladesh [3,4]. Like other countries, in Bangladesh inclusion of low-income people, particularly informal sector workers, for achieving UHC appears to be a challenge as this group is very mobile, un-organized, geographically dispersed and even are not tracked in a formal way through national registration system [5,6,7]. Furthermore, such informal workers alone constitute 88% of the total labor force in Bangladesh and contribute to 64% of total GDP [8], therefore, according to their contribution and volume, it is essential to make an effort to accumulate in the shade of a self-financed health scheme. The cooperative-based health insurance model can be a strong entity for reaching a large number of informal sector workers for developing self-financed community-based health scheme [9,10] and such concept are important for Bangladesh, as approximately 19.0 million households are connected with the informal sector, hence the potential scalability scope of such schemes [6]. Community-based health insurance is recognized as a promising tool of health system improvement for low-income people and improves the health status of enrolees and enhances productivity and labor supply [6,11]. However, securing health enrolment is critical for sustaining such scheme and many factors including insurance scheme design features such as benefit package, inflexible payment schedules and lack of awareness and clients’ satisfaction has a crucial role for the successful implementation of such scheme [6,12,13,14,15].

Recently the Healthcare Financing Strategy of Bangladesh emphasized the community-based health insurance, micro health insurance or other innovative initiatives as forms of social protection which are being tested in different parts of the country and those are still in their early stage of implementation [6,16]. However, none of the studies focused on benefit packages as well as client experiences and satisfactions toward health care services organized by this scheme although clients’ satisfactions are as important measures of health system performance [17,18]. Patient satisfaction is an important indicator of health care quality and often associated with greater adherence to medical technology, health service utilization, and health outcomes [19,20,21]. Huy and colleagues observed that satisfaction has a significant impact on patient retention, patient loyalty and influences the efficient delivery of quality in health care [22]. Furthermore, clients’ experience and opinions are important for improving healthcare services, shaping health policies and providing feedback on the quality, availability, and responsiveness of healthcare services. Therefore, studies should focus on clients’ satisfaction to ensure the quality of healthcare services provided by the health insurance scheme which are currently not available in this context. To address this information gap, this study tried to measure the degree of clients’ satisfaction towards healthcare services based on their experience of utilizing health care and factors associated with the satisfaction which will serve as the future reference point to implement potential quality improvement initiatives of community-based health insurance program in similar country context.

## 2. Materials and Methods

### 2.1. Study Settings and Sample

The study was conducted in a sub-district of Chandpur in Bangladesh where a community-based health insurance (CBHI) pilot program was carried out through a cooperative named Labor Association for Social Protection (LASP). The sub-district consists of 14 unions and a municipality. Seven unions located close to the municipality and the municipality itself were included as study sites. The process of enrolment in the CBHI scheme was voluntary and limited to informal sector workers. Workers with informal employment (including self-employment) were targeted by the scheme. There were two major criteria for enrollees: first, the household with income within the lowest taxable income bracket and, second, household with at least one blue-collar informal worker. The premium and associated benefits package are described below in detail:

Premium and benefits package of CBHI: Against one membership, a maximum of six household members were eligible to get access to CBHS benefit package, with a possibility of inclusion of more household member with additional fees. The premium was 600 BDT (7.72 USD) per household per year which was collected on monthly, quarterly or yearly basis. The details of the benefits packages is presented in Table 1.

### 2.2. Research Design

A cross-sectional household survey was conducted within the catchment area of LASP during April–June 2014 to compare the evaluation of the health care services provided by LASP scheme. The detailed sampling and the inclusion and exclusion criteria have been described elsewhere [6]. For measuring client satisfaction, a total of 233 clients who utilized CBHI services within the three months preceding this survey were randomly selected and considered as the respondents for this study. It is important to notice that we included only those clients who utilized health services through the insurance scheme for this study.

### 2.3. Data Collection

A structured questionnaire was developed and administered through face-to-face interview with household’s head/economic contributor or the household member of insured household who had experience and utilized the healthcare services provided by the healthcare service provider. The questionnaire included details on demographic characteristics of individual members and household socioeconomic characteristics, healthcare utilization and satisfaction related questions towards the current CBHI services. For healthcare service utilization and satisfaction we considered the CBHI beneficiaries who utilized LASP services within three months preceding this survey. A research team from icddr,b, an international health research organization, provided technical support for capacity building in collaboration with the Sajida Foundation, a micro-credit and health institute. All data collection tools were developed on the light of literature and pretested for quality control and field interviewers and supervisors were trained and supervised by field investigators. Training of the interviewers mainly focused on detailed data collection process with discussion of each question, what should be reported and how to avoid incorrect reporting as the paper-based questionnaire was editable. Difficulties of understanding and language barriers are a common phenomenon in household survey, therefore, significant training was provided to the data collectors with the local language. Piloting was done to identify potential barriers and to test the skills achieved by the data collectors. Filled questionnaire were discussed with the supervisors and team members to resolve the issues before final data collection. Data quality checking was the main role of the supervisors to ensure collection of the quality data and solving any issues if arose in data collection process. The data collectors explained the study objectives to the respondents before interviewing them and respondents were assured of their rights to withdraw from this study. The supervisors were not directly involved with the data collection process and, as such, they had no potential influence on the respondents and data collectors. The participation rate was 100% and informed written consent was obtained before conducting any given interview.

### 2.4. Data Analysis

In the descriptive analyses, the characteristics of the study participants were presented regarding frequency (*n*) and percentages (%) with 95% confidence interval (CI). In this study, Cronbach’s α was employed to test the internal consistency and reliability of the questionnaire for each domain. Spearman correlation analysis was performed between the satisfaction score of each indicator and overall satisfaction score. Based on the assumption of order or ranked data, Spearman correlation co-efficient showed more precise and reliable results compared with alternative methods (e.g., Pearson’s correlation). Multivariate linear regression analysis was used to identify factors associated with overall satisfaction towards the health scheme [17,23]. We estimated overall satisfaction score using Likert 5 scale [18] of beneficiary satisfaction of health scheme, for example, strongly disagree (1), disagree (2), neither agree nor disagree (3), agree (4) or strongly agree (5). Variables having *p*-value ≤ 0.05 in the bi-variate analysis were entered into multivariate regression models to control the effect of confounding. The variance inflation factor (VIF) test was done to determine whether multicollinearity was present or not. The Ramsey RESET test was performed to diagnose if there was any specification error of the model. Both unadjusted and adjusted co-efficient with 95% confidence interval (CI) were calculated to measure associations. For all the tests conducted in the study, a *p*-value of <0.05 was adopted as the statistically significant level. Data cleaning, validation, and all statistical analyses were done by using Stata/SE 13.0 (StataCorp LP, College Station, TX, USA).

### 2.5. Ethical Approval

The study protocol (PR-13007) was approved by the Institutional Review Board of the International Centre for Diarrhoeal Disease Research, Bangladesh (icddr,b). All respondents were given an information sheet in Bengali explaining their rights relating to their voluntary participation in the study and informed written consent were taken prior to interview.

## 3. Results

### 3.1. Background Characteristics

A total 233 CBHI beneficiaries utilized the health services within the last three months (Table 2). Among the total beneficiaries, male utilization was higher (65%) than that of female beneficiaries (35%). About 70% of beneficiaries were young adults (18–44 years), and 27% were middle-aged (45–64 years). Regarding the educational background, 41% of the beneficiaries had a medium educational attainment (secondary school level) followed by 37% of the participants who had a lower level of education (primary education or less). Only a small proportion (12%) of the participants had experienced higher secondary or higher level education. The majority of the participants were married (90%) and around half of the participant’s household had more than five members, followed by four to five members (43%). Among the total participants, 31% were housewives, and 22% were workers (e.g., rickshaw-pullers, hotel workers, van drivers), and the rest were self-employed, farmers, had informal employment and other. About 60% of participants had reported some or extreme health problems.

### 3.2. Distribution of Health Services Provision-Related Satisfaction on Health Scheme

Table 3 shows the health services provision-related satisfaction with the self-financed health scheme. The overall satisfaction mean score was 4.17 ± 0.04 (95% CI: 4.08–4.26) out of 5.0. The most satisfactory domains were related to the diagnostic services (4.46 ± 0.98), explanations about the prescribed medicines (4.23 ± 0.81), the surrounding environment of the healthcare facility (4.21 ± 0.70) and the behavior of health personnel toward clients (4.18 ± 0.73).

Participants to our question on satisfaction reported 68% were ‘very satisfied’ with diagnostic services as they believed that service provider of CBHI explained about the diagnostic tests properly, more than 50% of clients were ‘fairly satisfied’ with the friendly behaviour of service providers and staff, confidentiality of patient information, facility environment and about comprehensive health care services provided by the benefit packages (Table 3).

Regarding the characteristics of beneficiaries, the females were more satisfied (4.31 ± 0.65) with the overall scheme compared to males (Table 4). According to the overall degree of satisfaction level, a higher level of satisfaction score was observed among middle-aged (4.26 ± 0.64) and elderly (4.24 ± 0.65), low education (4.21 ± 0.59), small size of household (4.63 ± 0.75), housewife (4.29 ± 0.66) and worst health status (4.49 ± 0.74) beneficiaries. Univariate analyses (Table 4) identified significant positive associations (*p* < 0.05) between overall satisfaction with the health scheme and being female, extremely worst health status and economically disadvantaged households. However, the univariate analyses also detected negative associations between satisfaction and being a beneficiary living with four to five and more than five household members and being a businessman. The regression model explains 11.35% of the total variation (R^2^ = 0.1135). The Breusch–Pagan/Cook-Weisberg diagnostic test showed that heteroscedasticity was not present in the model. The VIF test with its mean (max) value of 3.78 (4.16) indicates that there is no evidence of a multicollinearity problem in the regression model [24]. The Ramsey RESET test showed that there is sufficient evidence against the hypothesis of omitted variable bias in the model.

In multivariate analyses (Table 4), among the socio-demographic variables, only worst self-reported health status of beneficiaries were significantly satisfied with health scheme (coefficient: 0.37, 95% CI: 0.09–0.65, *p* < 0.01). Likewise, sex, socioeconomic status, household size and occupation status were not significant (Table 4).

### 3.3. Standardized Satisfaction Items Test Scale

The overall Cronbach’s α (alpha) of the satisfaction domains was 0.93 (Table 5). Interestingly, the Cronbach’s satisfaction score was 0.92 for all of the satisfaction domains, which was significantly higher than the standard scale value (>0.72), i.e., clients were more satisfied with each of the services [17,23]. Furthermore, it signified that this was a positive indicator of internal consistency within each domain and the acceptable reliability of the satisfaction domains on the services provided by the health scheme.

## 4. Discussion

The World Health Organization has been advocating universal health coverage (UHC) so that all people who need health services should receive these services without facing financial hardship and should have full financial protection from out-of-pocket payment. According to the UHC theme, Bangladesh is also committed to achieving UHC by 2032. The Ministry of Health and Family Welfare of Bangladesh has currently conducted a pilot health scheme called Shasthyo Surokhsha Karmasuchi (SSK) targeting the most vulnerable households living below the poverty line. In the meantime, various community-based health insurance projects have been conducted in various parts of the country to reduce the excessive out-of-pocket payment and for securing sustainable quality healthcare coverage [6,11,25,26].

Despite the proven effects of community-based health insurance scheme, low enrolment in such schemes is critical and threatening the financial sustainability of the schemes in developing countries including Bangladesh [6,27,28,29,30,31]. There are various factors such as age, education, households’ head, household size, awareness about CBHI, self-related health, economic status, health provider characteristics which are often affect the CBHI enrolment [6,31,32,33]. Furthermore, perceived quality of health care services is the most vital single factor directly linked with participating in CBHI in many settings [33,34].

However, quality of healthcare services and health insurance often closely inter-related and service readiness is a key component to achieve UHC [31,35]. Thus assessing client satisfaction has an important role in designing future health insurance program and to promote client-oriented health services. This study explored clients’ satisfaction towards a community-based health insurance program organized by the informal sector workers in Bangladesh.

Our results observed that the overall score of satisfaction level was 4.17 out of 5 which means that the clients were highly satisfied with the health services provided by the self-financed health scheme. The overall Cronbach’s α (alpha) of the satisfaction domains was also high (0.93 out of 1.0) which ensured the most satisfaction with services of the scheme. The earlier study observed that the higher patients’ satisfaction is directly linked with the commitment of healthcare which often leads the better health outcome [36,37]. We found most of the clients were satisfied with the reception of the services and welcomed as they expected. They were also satisfied with the behavior of staff (e.g., paramedics, doctors) and the service providers (e.g., a specialized doctors, clinics, diagnostic centre) contracted out by the health scheme. Previous studies indicated that providers’ behavior towards the patients are directly linked with patients’ satisfaction [38,39]. The study observed that the clients were also satisfied for the role of providers contracted out by the insurance scheme. Most of the clients (more than 80%) reported that the provider had explained the prescribed medicines properly and 70% of the clients mentioned that service providers clarified their diagnostic tests properly. A healthy interaction with patients and healthcare providers are often positively linked with patients expectations and associated healthcare experiences which also influenced the patients’ satisfaction level [40,41]. This is also vital for the caregivers as caregivers also like to see the emotional support offered by the healthcare providers during the time of their distress [42]. The earlier study observed that client’s satisfaction level is positively linked with the enrolment of health insurance scheme which could be enhanced by maintaining a better patient-doctor relationship [17]. It has been noted that the satisfied patients often utilize more the healthcare services and followed the treatment regime properly [21,43], however poor quality of healthcare is often considered as a major source of dissatisfaction, particularly in CBHI programs [31].

Our study showed that most of the clients were satisfied with the facility environment (e.g., facility environment, cleanliness) provided by the health scheme which is in a similar line of the findings of other studies where the hospital environment, cleanliness and process management have been recognized as crucial patient satisfaction factors and a better physical environment of a health facility yielded greater patient satisfaction and even led to a positive perception towards the healthcare providers [17,44,45]. However, the literature suggests that the physical environment is able to produce reactions of dissatisfaction level rather than increasing satisfaction level as the environmental contamination is directly linked with the healthcare-associated infection [44,46,47]. A recent public hospital-based study in this context observed the provision of better cleanliness is significantly associated with patients’ satisfaction level [38]. Like various other studies, we also observed that socio-demographic characteristics are less important for clients’ satisfaction level towards providers’ services [17,48,49,50]. Our unadjusted model found a negative relationship among satisfaction and household size and occupations, although we could not establish the causal relationship here, however, financial issues might be one of the reasons as larger families required more healthcare and made frequent visits to the health facilities. Indeed most of the cases handled by income earner sof the households as costs involved which might be reflected their expectation towards healthcare and thus reduced satisfaction [50,51]. However, such a relationship was not observed at a significant level in our adjusted model. Our study observed that self-reported health status has a significant role in overall satisfaction towards healthcare services provided by the CBHI, which also supported by other studies [18,51,52]. We did not observe any significant relationship between economic status and the level of satisfaction although financial barriers often act as an important factor for accessing care and also the satisfaction level towards healthcare system [53,54]. However such relationship is not always clear, previous study indicated that uninsured people were less satisfied with the health-care system than insured population which was not investigated in this study [55]. Therefore more rigorous research is required to understand whether high patient satisfaction is associated with socio-economic and demographic factors as well as with the better health outcome.

This study has several limitations. First, the questionnaires were developed specifically for capturing the services provided by the health scheme with many contents tailormade and may not be a comprehensive survey for quality of care. Secondly, the design of the study was not randomized and we did not consider clients’ previous experience and perception about CBHI and therefore alternative explanations for client satisfaction ratings cannot be ruled out, and causal conclusions cannot be drawn. Thirdly, since all of the respondents belong to the health scheme this may bring bias in answering the questionnaire, therefore future research is needed to understand the determinants of satisfaction with the CBHI particularly the broader societal factors we could not explore in this analysis. The other limitations might involve the recall and incorrect reporting bias as the household survey was conducted after receiving the health care services, therefore, recall bias might jeopardize the study findings. Further, editable paper-based questionnaires may be another source of bias of the study. Furthermore, we conducted face to face interviews which is an effective method for data collection for primary research, however, biased responses could be delivered due to ‘anchor biased effect’ as their responses could be influenced by the interviewer. To avoid such limitations, proper training was provided by the project staff before collection of the data from the households. The other limitation is the sample size as the results were based on a cross-section survey within the LASP catchment area which covered only a sub-district level, therefore, the study might not be representative of the whole country. Despite these limitations, our study provides important findings on which future research on community-based health schemes can build.

## 5. Conclusions

The key findings from this paper are observations of client satisfaction with healthcare services provided by a community-based health scheme. Our study observed that the overall satisfaction level towards health services is quite favorable, but satisfaction scores can still be improved. These findings could contribute towards developing and designing the healthcare services packages of community-based health scheme which is in line with the health care financing strategy of Bangladesh as well as the recommendations of the World Health Organization for developing social health insurance as part of the path to Universal Health Coverage [56,57].

## Figures and Tables

**Table 1:** The benefits package of the CBHI.

Name of Services	Description of Services
Health benefits	
Qualified medical doctors consultation	30 BDT (US$0.39)—Market price 300 BDT (US$3.86)
Medicine/Drugs	20% discount on all medicines
Diagnostic tests	Up to 50% discount on all diagnostic tests
Specialist doctor consultation (e.g., gynaecologists, cardiologists)	100 BDT (US$1.29) (Market price = 500 BDT or US$6.44)
Inpatient care	Maximum 4000 BDT (US$51.45) per household per year
Periodic satellite clinics	Free of charge is LASP surveillance area
Non-health benefits	
Savings Opportunity	Up to 500 BDT (US$6.44) per month per household. Member can withdraw any saved amount with 10% interest after one year period
Low cost Computer Training Programs (CTP)	For all beneficiaries of LASP, with a cost 1200 BDT (US$15.44), Length of training was three months; market price 4500 BDT (US$58.0)
Sewing Training (ST)	For all female CBHI beneficiaries; Length of training was three months with free of charge

**Table 2:** Background characteristics of beneficiaries (*N* = 233).

Variables	Observation (*n*)	Percentage (%)
Sex		
Male	151	64.81
Female	82	35.19
Age		
18–44	160	68.67
45–64	64	27.47
≥65	9	3.86
Education background		
No education	23	9.87
Primary	87	37.34
Secondary	96	41.20
Higher secondary	20	8.58
Higher	7	3.00
Marital status		
Unmarried	16	6.87
Married	211	90.56
Widowed/divorced/Separated	6	2.58
Family size		
<4	11	4.72
4–5	99	42.49
>5	123	52.79
Occupations		
Worker	52	22.32
Business	40	17.17
Housewife	72	30.9
Farmer	14	6.01
Services	28	12.02
Other	27	11.59
Self-reported health states		
No problem	92	39.48
Some problems	103	44.21
Extreme problems	38	16.31
Income quintile (BDT)		
Q1 (≤8000 BDT)	48	20.61
Q2 (8001–12,000 BDT)	62	26.61
Q3 (12,001–15,000 BDT)	34	14.59
Q4 (15,001–24,000 BDT)	43	18.45
Q5 (>24,000 BDT)	46	19.74
Total observation, *N*	233	100.00

**Table 3:** Health services provision-related satisfaction on self-financed health scheme.

Domain	Likert 5 Scale of Patient Satisfaction	*n* (%)	Score	
			Mean ± SD	95% CI
Satisfied with reception of services (*N* = 233)	(1) Not good	11 (4.72)	3.88 ± 1.10	3.74 to 4.02
	(2) A little bit	13 (5.58)		
	(3) Quite good	51 (21.89)		
	(4) Welcomed nicely	76 (32.62)		
	(5) Welcomed greatly	82 (35.19)		
Satisfied with service providers’ attitude towards explaining health problem (*N* = 233)	(1) Strongly disagree	3 (1.29)	4.08 ± 0.93	3.96 to 4.20
	(2) Disagree	10 (4.29)		
	(3) Neutral	44 (18.88)		
	(4) Agree	84 (36.05)		
	(5) Strongly agree	92 (39.48)		
Services providers friendly (*N* = 233)	(1) Strongly disagree	2 (0.86)	4.18 ± 0.79	4.08 to 4.28
	(2) Disagree	4 (1.72)		
	(3) Neutral	32 (13.73)		
	(4) Agree	107 (45.92)		
	(5) Strongly agree	88 (37.77)		
Service providers’ explanations about prescribed medicines (*N* = 233)	(1) Not explained simply	3 (1.29)	4.23 ± 0.81	4.12 to 4.33
	(2) Partly explained	5 (2.15)		
	(3) Fairly explained	23 (9.87)		
	(4) Has been explained	107 (45.92)		
	(5) Been fully explained	95 (40.77)		
Service providers’ explanations about diagnostic tests (*N* = 149)	(1) Not explained simply	6 (4.03)	4.46 ± 0.98	4.31 to 4.62
	(2) Partly explained	2 (1.34)		
	(3) Fairly explained	10 (6.71)		
	(4) Has been explained	30 (20.13)		
	(5) Been fully explained	101 (67.79)		
Satisfied with confidentiality (*N* = 233)	(1) Strongly disagree	6 (2.58)	4.09 ± 0.86	3.97 to 4.20
	(2) Disagree	5 (2.15)		
	(3) Neutral	26 (11.16)		
	(4) Agree	122 (52.36)		
	(5) Strongly agree	74 (31.76)		
Satisfied with staff behaviour (*N* = 233)	(1) Strongly disagree	2 (0.86)	4.18 ± 0.73	4.09 to 4.28
	(2) Disagree	2 (0.86)		
	(3) Neutral	27 (11.59)		
	(4) Agree	122 (52.36)		
	(5) Strongly agree	80 (34.33)		
Satisfied with facility environment (*N* = 233)	(1) Strongly disagree	-	4.21 ± 0.70	4.12 to 4.30
	(2) Disagree	1 (0.43)		
	(3) Neutral	34 (14.59)		
	(4) Agree	114 (48.93)		
	(5) Strongly agree	84 (36.05)		
Satisfied with the comprehensive services provided by the clinic (*N* = 233)	(1) Strongly disagree	3 (1.29)	4.09 ± 0.85	3.98 to 4.20
	(2) Disagree	8 (3.43)		
	(3) Neutral	32 (13.73)		
	(4) Agree	112 (48.07)		
	(5) Strongly agree	78 (33.48)		
Overall score			4.17 ± 0.04 (4.08 to 4.26)	

**Table 4:** Distribution of health services provision-related average satisfaction score on self-financed health scheme across the beneficiary characteristics.

Variables	Mean Satisfaction Score ± SD	Unadjusted Model			Adjusted Model		
		Cofficient (SE)	95% CI	*p*-Value	Coefficient (SE)	95% CI	*p*-Value
Sex							
Male	4.09 ± 0.67	Ref	-	-	Ref	-	-
Female	4.31 ± 0.65	0.22 (0.09)	(0.04, 0.40)	0.01	0.20 (0.24)	(−0.28, 0.68)	0.41
Age							
18–44	4.13 ± 0.68	Ref	-	-			
45–64	4.26 ± 0.64	0.14 (0.10)	(−0.05, 0.33)	0.16			
≥65	4.24 ± 0.65	0.11 (0.21)	(−0.31, 0.53)	0.62			
Education background							
No education	4.21 ± 0.59	0.34 (0.43)	(−0.51, 1.19)	0.43			
Primary	4.17 ± 0.69	0.30 (0.42)	(−0.53, 1.13)	0.47			
Secondary	4.19 ± 0.66	0.32 (0.42)	(−0.51, 1.14)	0.45			
Higher secondary	4.11 ± 0.55	0.24 (0.43)	(−0.61, 1.09)	0.59			
Higher	3.88 ± 1.17	Ref	-	-			
Marital status							
Unmarried	4.21 ± 0.58	Ref	-	-			
Married	4.15 ± 0.68	−0.06 (0.17)	(−0.40, 0.29)	0.74			
Widowed/divorced/Separated	4.58 ± 0.47	0.37 (0.32)	(−0.26, 1.00)	0.25			
Family size							
<4	4.63 ± 0.75	Ref	-	-	Ref	-	-
4–5	4.19 ± 0.71	−0.43 (0.21)	(−0.85, −0.02)	0.04	−0.31 (0.26)	(−0.83, 0.2)	0.23
>5	4.11 ± 0.62	−0.51 (0.21)	(−0.93, −0.10)	0.01	−0.35 (0.26)	(−0.87, 0.16)	0.18
Occupations							
Housewife	4.29 ± 0.66	Ref	-	-	Ref	-	-
Worker	4.23 ± 0.60	−0.07 (0.12)	(−0.31, 0.17)	0.59	0.11 (0.25)	(−0.38, 0.61)	0.65
Businessman	4.03 ± 0.62	−0.26 (0.13)	(−0.52, 0.00)	0.05	0.01 (0.26)	(−0.49, 0.52)	0.97
Farmer	4.08 ± 0.62	−0.21 (0.20)	(−0.59, 0.18)	0.29	0.01 (0.3)	(−0.58, 0.59)	0.99
Services	4.06 ± 0.70	−0.23 (0.15)	(−0.52, 0.06)	0.12	−0.04 (0.28)	(−0.6, 0.51)	0.88
Other	4.10 ± 0.88	−0.19 (0.15)	(−0.49, 0.11)	0.20	−0.13 (0.23)	(−0.58, 0.33)	0.58
Self-reported health states							
No problem	4.03 ± 0.62	Ref	-	-	Ref	-	-
Some problems	4.18 ± 0.65	0.14 (0.09)	(−0.04, 0.33)	0.13	0.11 (0.1)	(−0.09, 0.31)	0.27
Extreme problems	4.49 ± 0.74	0.45 (0.13)	(0.2, 0.7)	0.00	0.37 (0.14)	(0.09, 0.65)	0.01
Income quintile (BDT)							
Q1 (≤8000 BDT)	4.00 ± 0.56	0.24 (0.14)	(−0.03, 0.51)	0.08	0.05 (0.15)	(−0.25, 0.34)	0.75
Q2 (8001–12,000 BDT)	4.24 ± 0.72	0.35 (0.13)	(0.10, 0.61)	0.01	0.23 (0.14)	(−0.06, 0.51)	0.12
Q3 (12,001–15,000 BDT)	4.35 ± 0.71	0.08 (0.15)	(−0.21, 0.38)	0.59	0 (0.15)	(−0.3, 0.3)	0.99
Q4 (15,001–24,000 BDT)	4.08 ± 0.69	0.10 (0.14)	(−0.18, 0.38)	0.49	0.06 (0.14)	(−0.21, 0.33)	0.68
Q5 (>24,000 BDT)	4.09 ± 0.61	Ref	-	-	Ref	-	-
Observation (N)						233	
VIF Mean (Max)						3.78 (4.16)	
Ramsey RESET test						5.49 (<0.001)	
R-squared						11.35%	
F-statistic						2.38 (<0.004)	

**Table 5:** Standardized satisfaction items test scale on self-financed health scheme.

Domain	*N*	Sign	Item-Test Correlation	Item-Rest Correlation	Inter-Item Correlation	Alpha
Satisfied with reception of services	233	+	0.8	0.74	0.61	0.93
Satisfied with service providers attitude towards explaining health problem	233	+	0.84	0.79	0.6	0.92
Services providers friendly	233	+	0.87	0.83	0.59	0.92
Service providers explaining about prescribed medicine	233	+	0.84	0.79	0.6	0.92
Service providers explanation about diagnostic tests	149	+	0.64	0.54	0.63	0.93
Satisfied with confidentiality	233	+	0.8	0.74	0.61	0.92
Satisfied with staff behavior	233	+	0.85	0.8	0.59	0.92
Satisfied with facility environment	233	+	0.74	0.67	0.62	0.93
Satisfied with clinic provides comprehensive services	233	+	0.82	0.76	0.6	0.92
Test scale					0.61	0.93
